# Mesenteric Lymphadenitis Presenting as Acute Abdomen in a Child with Multisystem Inflammatory Syndrome

**DOI:** 10.3390/idr14030046

**Published:** 2022-06-06

**Authors:** Evangelos Blevrakis, Eleni Vergadi, Maria Stefanaki, Iris Alexiadi-Oikonomou, Glykeria Rouva, Ioannis Germanakis, Emmanouil Galanakis

**Affiliations:** 1 Department of Paediatric Surgery, School of Medicine, University of Crete, 715 00 Heraklion, Greece; e.blevrakis@uoc.gr (E.B.); maria.stef.99@hotmail.com (M.S.); iridaa99@gmail.com (I.A.-O.); 2 Department of Paediatrics, Medical School, University of Crete, 715 00 Heraklion, Greece; kelrouva@gmail.com (G.R.); yannis.germanakis@gmail.com (I.G.)

**Keywords:** MIS-C, COVID-19, SARS-CoV-2, acute abdomen, pediatric surgery, exploratory laparotomy, mesenteric adenitis

## Abstract

Multisystem inflammatory syndrome in children (MIS-C) may develop as a rare complication following COVID-19. MIS-C presentation varies substantially, but fever and gastrointestinal symptoms are the most prominent. Indeed, gastrointestinal involvement may be severe enough to present as acute abdomen, posing challenges to clinicians. We present herein the case of a healthy five-year-old male who presented with fever, vomiting, and abdominal pain, resembling acute abdomen. The patient had no history of SARS-CoV-2 infection or exposure, and MIS-C diagnosis was initially surpassed unnoticed. The patient underwent exploratory laparotomy that only revealed mesenteric lymphadenitis. Postoperatively, the patient met the clinical and laboratory diagnostic criteria of MIS-C. SARS-CoV-2 exposure was serologically confirmed and MIS-C treatment was commenced, resulting in defervescence and a satisfactory outcome. In young patients presenting with acute abdomen, surgeons should be aware of MIS-C, so that earlier diagnosis and appropriate treatment are made prior to surgical interventions.

## 1. Introduction

Coronavirus disease (COVID-19) may cause a wide range of clinical manifestations, from asymptomatic cases to severe acute respiratory distress syndrome, and death [1]. Children usually present with a milder course of infection or can even act as asymptomatic carriers of SARS-CoV-2, having a more favorable prognosis compared to adults [2].

However, an increasing number of reports have highlighted a rare complication following COVID-19 in children, sharing similar characteristics with Kawasaki disease, the multisystem inflammatory syndrome in children (MIS-C) [3,4]. MIS-C is a hyperinflammatory state, defined as a constellation of the following criteria; age less than 21 years old, fever > 38 °C for over 24 h, severe illness requiring hospitalization with dysfunction of at least two organ systems, laboratory evidence of inflammation, laboratory or epidemiologic evidence of current or recent infection or exposure to SARS-CoV-2, and no alternative plausible diagnosis [4,5,6].

The clinical presentation of MIS-C varies substantially, but gastrointestinal manifestations are among the most prominent [7]. Gastrointestinal (GI) symptoms such as abdominal pain, vomiting, and diarrhea are commonly reported, and GI involvement may be severe enough to resemble acute abdomen, posing challenges to clinicians [7]. We present herein the case of a child who presented with MIS-C and acute abdomen and underwent emergency laparotomy before a definitive diagnosis was made. Laparotomy was suggestive of mesenteric lymphadenitis, and MIS-C diagnosis was only made postoperatively when the patient met the clinical and laboratory diagnostic criteria of MIS-C.

## 2. Case Presentation

A previously healthy five-year-old boy, with no previous history of SARS-CoV-2 infection, was referred to our pediatric surgical department in May 2021. Initially, the boy presented to the emergency department of a regional hospital with a two-day history of fever (40 °C) and a one-day history of diffuse abdominal pain, vomiting, and diarrhea. At admission, a nasopharyngeal PCR for SARS-CoV-2 was performed and he tested negative. Laboratory tests showed normal white blood cells (WBCs) (7310/μL), with a neutrophilic predominance (93%), hemoglobin value of 12.4 g/dL, platelets (PLT) 196,000/μL, and C-reactive protein (CRP) 10.9 mg/dL (normal range; 0–0.5 mg/dL). Stool cultures were negative, and urinalysis was normal. On the second day of hospitalization, the boy showed signs of abdominal flatulence. Dilated bowel loops were present and the stepladder sign at abdominal X-ray. In addition, oedema of the lower extremities and upper eyelids was noted. An abdominal ultrasound was performed that revealed a considerable amount of free fluid in the upper and lower abdomen. Chest X-ray was clear. Cefotaxime and metronidazole were administered, but fever persisted.

**Figure 1:**
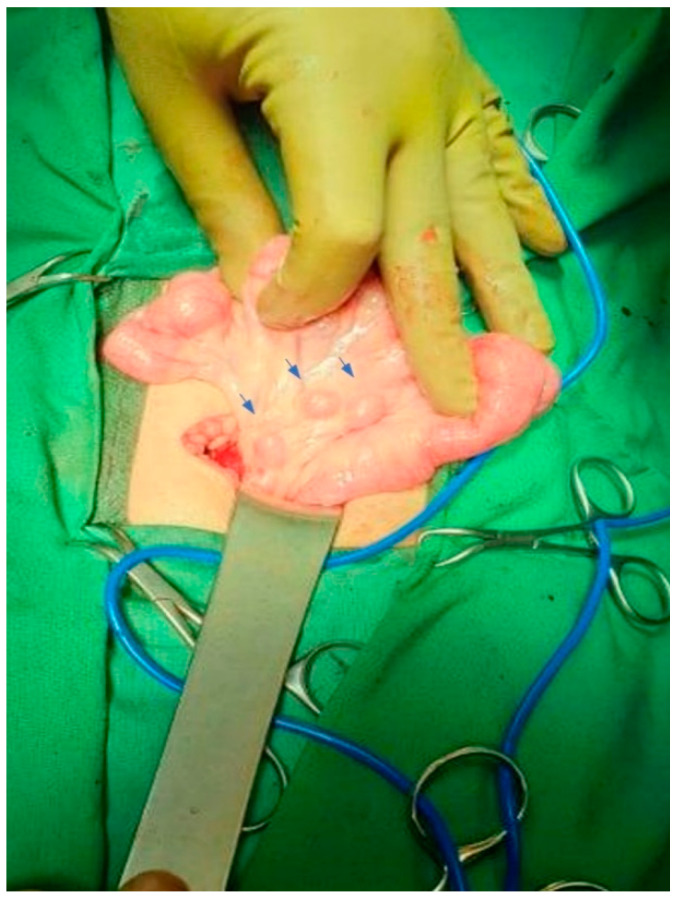
Multiple enlarged mesenteric lymph nodes (arrows) revealed during exploratory laparotomy.

Six days after the onset of the symptoms, due to the persistence of fever and abdominal pain, the boy was referred to our tertiary hospital for surgical consultation. Acute abdomen was suspected, and exploratory laparotomy was performed. A significant amount of free fluid, not pus, was found in the peritoneal cavity, along with multiple enlarged mesenteric lymph nodes (Figure 1), without signs of acute appendicitis. Due to the absence of any surgical pathology, the boy was transferred to the pediatric department for further evaluation.

On physical examination postoperatively, the child appeared febrile with a temperature of 38.5 °C, irritable, and pale. Tachycardia with gallop rhythm and tachypnea were present. The lower extremity and upper eyelid edema persisted. Additionally, an erythematous, spotty, non-itchy skin rash on the shins, ankles, thighs, back, and face was noted. Laboratory findings revealed normal WBCs (10,100/μL) with neutrophilic predominance (85%), lymphopenia (1000/uL), thrombocytopenia (99,000/μL), hypoalbuminemia (3 g/dL), elevated inflammatory markers (CRP 9.7 mg/dL, interleukin-6 39.3 pg/mL, normal range <6, and erythrocyte sedimentation rate of 50 mm/h), coagulopathy with elevated D-dimers (3.7 mg/dL, normal range <0.5 mg/dL), and abnormal cardiac function as was reflected by elevation of troponin (hsTnI 54.1 pg/mL, reference range <11.6), and B-natriuretic peptide (BNP 643 pg/mL, reference range <100). Cultures of intraperitoneal fluid, blood, urine, stool, and pharyngeal swab came out sterile. An electrocardiogram was performed and showed ST wave elevation and T wave inversion, suggesting myocardial involvement.

Due to the presence of multisystem involvement, such as the skin (rash), GI symptoms (vomiting, diarrhea), cardiac involvement, and coagulopathy, MIS-C was suspected. SARS-CoV-2 antibody testing was performed that came out positive (IgM 1.28, IgG 24, positive values >1), indicating prior SARS-CoV-2 infection. Further evaluation with echocardiography reflected mild left ventricle and left atrium dilation with good systolic function, mild to moderate mitral regurgitation, small pericardial effusion, as well as increased echogenicity and dimensions of coronary arteries, especially left and right coronary arteries, with z-scores of 2.5.

Postoperatively, oxygen supplementation and intravenous hydration were administered, and furosemide and human albumin substitution were added to manage oedema. Once MIS-C diagnosis was established, immunotherapy with intravenous immunoglobulin (IVIG 2 g/kg/d within 12 h) and methylprednisolone (2 mg/kg/d) was initiated. The child also received anti-platelet therapy with low-dose aspirin. Treatment resulted in remarkable improvement with defervescence, remission of the skin rash, and extremities eodema. At the 14-day follow-up, his clinical status was excellent and the cardiac complications as well as coronary arteries z-scores, assessed by repeated echocardiography, returned to normal.

## 3. Discussion

MIS-C, first reported in April 2020, is currently a well-established post-COVID-19 complication and is considered to be related to a dysregulated immune response to infection [4]. SARS-CoV-2-induced endothelial dysfunction and cytokine storm have been proposed as pathogenetic mechanisms of MIS-C [8,9].

As of now, gastrointestinal symptoms are the most prominent in MIS-C, occurring in nearly 92% of patients, followed by cardiac involvement in 80% [5,7]. These GI symptoms can mimic many other infections and inflammatory diseases in children, including acute abdomen, as in our case. Apart from fever and GI symptoms, other typical symptoms of MIS-C include skin rash, conjunctivitis, lymphadenopathy, mucosal changes, oedema, coronary artery dilatation or aneurysms, myocarditis, pneumonia, and neurologic symptoms of variable severity as well as raised inflammatory markers, lymphopenia, and thrombocytopenia [4,6,10,11].

Our case presented with the majority of the typical clinical manifestations of MIS-C, such as persistent fever, GI symptoms, lymphopenia, thrombocytopenia, elevated pro-BNP, and CRP. However, all the above symptoms were not evident at initial presentation. Additionally, the patient’s family denied any previous infection or known exposure to SARS-CoV-2. Given both imaging and laboratory results and his severe clinical presentation, the decision for exploratory laparotomy was made. In our case, the presence of acute abdomen leads to prompt surgical evaluation prior to MIS-C diagnosis. The operation was proven unnecessary, as no surgical pathology was discovered. Indeed, in the literature, there are many cases of MIS-C and acute abdomen, in which laparotomy was performed and was proven to be unnecessary [7]. As in our case, mesenteric lymphadenitis was the most prominent finding in these cases [7]. Interestingly, it has been shown that imaging studies, such as abdominal ultrasound or abdominal computed tomography (CT), may facilitate in distinguishing true surgical emergencies in cases with MIS-C and acute abdomen [7]. In our case, abdominal ultrasound was not helpful. Abdominal CT was not performed, and whether the performance of an abdominal CT will have prevented us from performing a laparotomy is unknown.

Postoperatively, the negative laparotomy, in conjunction with the skin rash, GI symptoms, and elevated inflammatory markers, raised clinical suspicion of MIS-C and prompted serology testing, the positive outcome of which confirmed the diagnosis. Conversely to our case which mimicked surgical abdomen, surgically confirmed cases of acute appendicitis have been described in patients with MIS-C [12,13,14,15,16]. Cases of intestinal ischemia and acute pancreatitis have been also reported [17,18]. Resembling our case, laparoscopic appendectomy was performed in a four-year-old girl with recent exposure to SARS-CoV-2, with the appendix appearing grossly normal [19]. The non-operative treatment of uncomplicated acute appendicitis and concomitant COVID-19 infection with antibiotics has been also reported [20].

This case strengthens the association between SARS-CoV-2, MIS-C, and GI symptoms. As COVID-19 cases in children continue to rise, our objective is to emphasize the need of maintaining a broad differential diagnosis, especially in paediatric patients presenting with acute abdomen and prolonged fever with elevated inflammatory markers, even in the absence of a history of SARS-CoV-2 infection. Paediatric surgeons should maintain a high index of suspicion for MIS-C in patients with a clinical presentation mimicking a surgical abdomen, to avoid unnecessary surgical procedures.
